# Terahertz near-field microscopy based on an air-plasma dynamic aperture

**DOI:** 10.1038/s41377-022-00822-8

**Published:** 2022-05-07

**Authors:** Xin-ke Wang, Jia-sheng Ye, Wen-feng Sun, Peng Han, Lei Hou, Yan Zhang

**Affiliations:** 1 Beijing Key Laboratory of Metamaterials and Devices, Key Laboratory of Terahertz Optoelectronics Ministry of Education, Department of Physics, Capital Normal University, Beijing, 100048 China; 2 Applied Physics Department, Xian University of Technology, Xian, Shaanxi 710048 China

**Keywords:** Imaging and sensing, Terahertz optics

## Abstract

Terahertz (THz) near-field microscopy retains the advantages of THz radiation and realizes sub-wavelength imaging, which enables applications in fundamental research and industrial fields. In most THz near-field microscopies, the sample surface must be approached by a THz detector or source, which restricts the sample choice. Here, a technique was developed based on an air-plasma dynamic aperture, where two mutually perpendicular air-plasmas overlapped to form a cross-filament above a sample surface that modulated an incident THz beam. THz imaging with quasi sub-wavelength resolution (approximately *λ*/2, where *λ* is the wavelength of the THz beam) was thus observed without approaching the sample with any devices. Damage to the sample by the air-plasmas was avoided. Near-field imaging of four different materials was achieved, including metallic, semiconductor, plastic, and greasy samples. The resolution characteristics of the near-field system were investigated with experiment and theory. The advantages of the technique are expected to accelerate the advancement of THz microscopy.

## Abstract (web summary)

A THz near-field technique was proposed based on an air-plasma dynamic aperture, which can achieve sub-wavelength THz imaging without approaching the sample with any devices.

## Graphical abstract

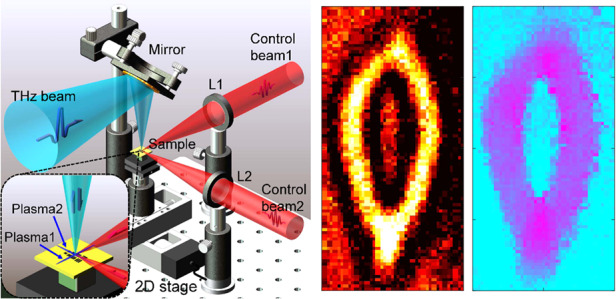

## Introduction

Because of the unique properties of terahertz (THz) radiation, such as non-ionizing photon energies, high transmittance for non-polar materials, and broad spectral information, the development, and applications of THz imaging have attracted considerable attention^1,2^. Unfortunately, the resolution of THz imaging is always limited to the millimeter-scale because of its long wavelength (1 THz–300 µm). Since 1998, when Hunsche et al. introduced a metallic aperture into a THz imaging system to achieve sub-wavelength resolution^3^, THz near-field microscopy has been rapidly developed^4^. Numerous methods have been introduced to optimize its performance, such as tips used for atomic force or scanning tunneling microscopy^5–7^, micro-antenna probes^8,9^, and dielectric spheres or cubes with high numerical apertures^10,11^. In 2000, Chen et al. utilized a photoexcited semiconductor wafer for THz sub-wavelength imaging and proposed the concept of a dynamic aperture^12^. The method greatly expanded the possibility of improving THz near-field microscopy. In 2017, Stantchev et al. combined this method with a compressed sensing technique to achieve an imaging resolution of 9 µm^13^. In 2020, Chen et al. adopted a spintronic THz-emitter-array to replace the semiconductor wafer and achieved THz ghost imaging with deep sub-wavelength resolution^14^.

Currently, the applications of THz near-field microscopy are mainly focused on fundamental investigation of condensed matter systems, such as ultrafast control of current on an atomic scale^15^, imaging of hot-electron energy dissipation^16^, and characterization of atomic-scale field transients^17^. In all these current THz near-field techniques, however, it has been necessary to approach the sample surface with a THz detector or source. This problem has restricted the wider applications of THz near-field microscopy in other fields, e.g., biomedical sensing and chemical inspection. Numerous researchers have demonstrated that normal and diseased tissues can be identified via THz imaging^18,19^, and that different chemicals can also be distinguished using THz imaging^20,21^. Undoubtedly, the introduction of THz near-field microscopy could enhance the measurement accuracy of THz techniques greatly in these fields. Unfortunately, the samples in applications of this type are generally soft and uneven, and this means that they are difficult to measure when using conventional THz near-field techniques. Therefore, there is an urgent need to develop a suitable THz near-field technique.

Air-plasmas have been widely investigated as THz emitters^22–24^ and detectors^25–27^. Under the combined actions of geometrical focusing, diffraction effects, Kerr self-focusing, and plasma defocusing, air-plasmas can form filaments with millimeter- or centimeter-scale lengths and micron-scale diameters^28^. In 2014, Zhao et al. used a two-color air-plasma as a THz emitter to realize imaging with 20 µm resolution^29^. In 2015, Buccheri and Zhang investigated the THz emission properties of a micro-plasma and pointed out its potential applications in THz microscopy^30^. However, a sample surface can be easily damaged if it is directly exposed to an air-plasma. Hence, they have been problematic for THz microscopy.

Here, we developed a new approach for THz near-field microscopy based on an air-plasma dynamic aperture. A cross-filament was formed by two crossed air-plasmas, which opened a dynamic aperture to modulate the intensity of a THz beam on a sample surface. The cross-filament was close enough to the sample surface to produce quasi sub-wavelength resolution. The main advantage of this technique was that no actual THz detector or source approached the sample, and surface damage from the cross-filament was minimized. Four types of samples were tested, including a metallic resolution test chart, a semiconductor chip, a plastic pattern, and a greasy spot. The resolution was investigated in detail and a physical model was used to explain the mechanism. It is anticipated that the technique will play a crucial role in THz applications, such as biomedical sensing and chemical inspection.

## Results

### Concept design

**Fig. 1:**
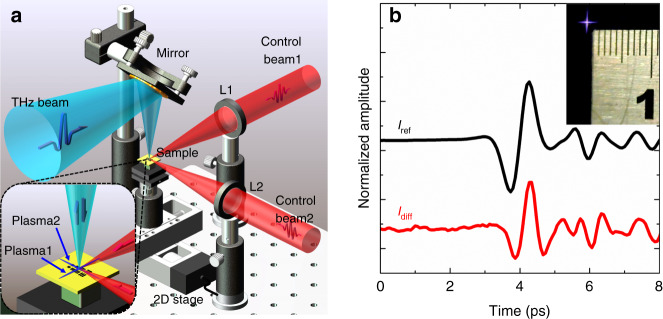
Design concept. **a** Schematic of terahertz (THz) near-field microscopy based on an air-plasma dynamic aperture. Two femtosecond laser pulses were focused in mutually perpendicular directions to generate two air-plasmas (Plasma1 and Plasma2) close to the sample surface. The incident THz beam was modulated by the cross-filament created by the air-plasmas and the reflected THz near-field signal was measured. The inset shows the relationships between the two air-plasmas, the THz beam, and the sample. **b** Normalized reference (*I*_ref_) and near-field (*I*_diff_) THz temporal signals reflected by a metallic plate. The inset shows the cross-filament and a ruler

The basic operational concept of the air-plasma dynamic aperture is shown in Fig. 1a. Two femtosecond laser pulses (Control beam1 and Control beam2) were focused by two convex lenses (L1 and L2) to generate two air-plasmas (Plasma1 and Plasma2), respectively. With grazing incidence angles, the two air-plasmas separately propagated past the sample in mutually perpendicular directions. Their central regions overlapped to form a cross-filament very close to the sample surface, as shown in the inset of Fig. 1a. Because an air-plasma has high electron density^31^, it can produce strong absorption and reflection effects in the THz frequency range. Therefore, an air-plasma can be regarded as being analogous to a metallic needle for a THz beam. When a converging orthogonal THz beam irradiated the sample through the cross-filament, its intensity was modulated by the plasma screening effect^32^. Moreover, by using a double modulation scheme with two lock-in amplifiers, only the THz signal modulated by the central part of the cross-filament was detected. Because an air-plasma generally has a micron-scale diameter^33^, the cross-filament region of the two air-plasmas opened a micron-scale dynamic aperture for the THz beam. The sample was mounted on a two-dimensional motorized stage for raster scanning. Thus, evanescent THz field information was detected and a THz image of the sample was obtained with sub-wavelength spatial resolution. As noted above, the air-plasma did not directly impinge on the sample surface, avoiding sample damage.

To verify the proposed concept, we constructed a THz imaging system that used a normal reflection measurement mode. In this system, a laser pulse was divided into a pump beam, a probe beam, and two control beams. The pump and probe beams were used to generate and detect THz radiation, respectively. The two control beams were focused in mutually perpendicular directions, and a cross-filament was formed above the sample. The THz signal that was reflected by the sample and modulated by the cross-filament was then detected using an electro-optic sampling method. Two mechanical choppers with different operating frequencies were inserted separately in the paths of the two control beams to modulate their outputs. Two lock-in amplifiers were used to implement the double modulation scheme required to extract the THz near-field signal (see sections I-a and I-b in the Supplementary Information (SI) for more experimental details).

A metallic plate was used to characterize the THz near-field signal modulated by the cross-filament. Normalized reference (*I*_ref_) and near-field (*I*_diff_) THz temporal signals were acquired in sequence, as shown in Fig. 1b. *I*_ref_ was the far-field THz signal reflected by the sample that was measured by using single modulation and a lock-in amplifier. In the far-field measurement, the two control beams were blocked and a mechanical chopper was used to modulate the THz beam (see section II-a in the SI). In the near-field measurement, the pulse energies of the two control beams were both 0.3 mJ and the L1 and L2 focal lengths were both 15 cm. The diameters of the two incident control beams were limited to 7 mm. The height *h* of the cross-filament above the sample surface was adjusted to approximately 50 µm. With the above parameters, a modulation depth of the THz beam by the cross-filament was ensured and sample damage was avoided. The signal-to-noise ratio (SNR) of *I*_diff_ was significantly less than that of *I*_ref_, because only a small portion of the THz signal was detected in the near-field. The temporal evolution of *I*_diff_ was similar to that of *I*_ref_, but deviations indicated that the modulation of the THz beam by the cross-filament was dispersive (see section I-c in the SI). In addition, the plasma fluorescence profile of the cross-filament was acquired with a charge-couple device camera, as shown in the inset of Fig. 1b. The central region of the cross-filament was tens of square microns.

### Imaging capability

**Fig. 2:**
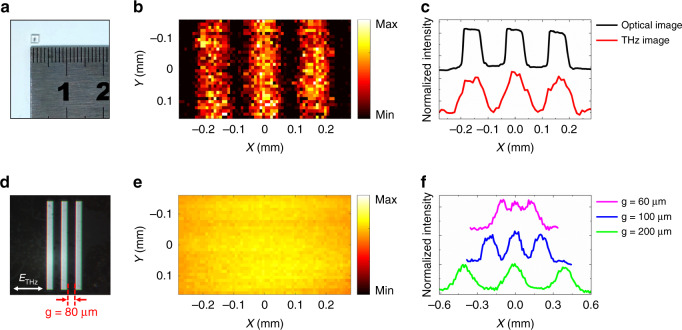
Imaging capability. **a** Metallic resolution test chart and a ruler. The chart had three chromium (Cr) stripes on a glass substrate that were separated by two 80 µm slit widths. **b** Near-field image of the chart acquired at 1.35 THz. **c** Normalized THz and optical intensity profile curves extracted from b and d along the *X* direction. **d** Magnified view of the chart, obtained with an optical microscope. The orientation of the THz polarization is also given. **e** Far-field image of the chart acquired at 1.35 THz. **f** Normalized intensity profiles extracted from near-field images acquired at 1.35 THz of three other test charts having slit widths of 60, 100, and 200 µm

A metallic resolution test chart fabricated via photolithography was used as a sample to calibrate its THz near-field image. It featured three chromium (Cr) stripes spaced 80 µm apart on a glass substrate. The chart is shown in Fig. 2a, and a magnified optical microscope image is shown in Fig. 2d. The polarization of the incident THz beam was perpendicular to the Cr stripes, and the sample was raster scanned with a 10 µm step. The imaging region was 56 × 32 pixels. During imaging, *h* was approximately 50 µm as noted above, and the pulse energies of the control beams were both 0.3 mJ. At each scan point, the THz spectral intensity $\left| {E_{{{{\mathrm{THz}}}}}\left( v \right)} \right|^2$ was extracted as the image data by performing a Fourier transformation, where $E_{{{{\mathrm{THz}}}}}\left( v \right)$ was the THz spectral amplitude of frequency $v$. The THz near-field image of the test chart for the central frequency at *v* = 1.35 THz is shown in Fig. 2b, in which the sample morphology was clearly revealed. The normalized intensity profile along the *X* axis at *Y* = 0 mm is shown in Fig. 2c. Each pixel value was averaged from five pixels along the *Y* direction, with prior knowledge of the vertical homogeneity of the sample. For comparison, the optical intensity profile of the sample from Fig. 2d was also plotted in Fig. 2c. The two curves were very consistent. The central wavelength of the THz near-field signal modulated by the cross-filament was 222 µm (*v* = 1.35 THz; see section I-c in the SI), and fine structures on the 80 µm spatial scale of the sample could be discerned. The imaging capability of the technique was demonstrated. For comparison, the sample was also imaged in the THz far-field, as exhibited in Fig. 2e. Because the resolution in the far-field for 1.35 THz was approximately 3.2 mm (see section II-b in the SI), sample details could not be distinguished. Other test charts with spacings of 60, 100, and 200 µm were imaged via THz near-field microscopy, and their normalized intensity profiles for 1.35 THz were plotted in Fig. 2f. The 100 and 200 µm spacings were accurately imaged. The 60 µm spacing was beyond the resolution limit, but its morphology could still be roughly discerned (see section II-c in the SI).

### Resolution estimation

**Fig. 3:**
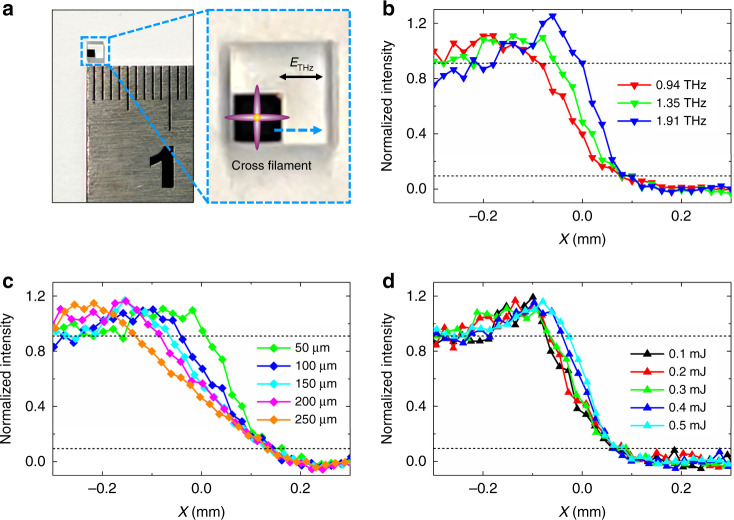
Resolution estimation. **a** Sample for the knife-edge method and a ruler. The sample was a 1 mm×1 mm Cr film on a glass substrate. The edge of the film was raster scanned along the *X* direction to evaluate the spatial resolution of the THz near-field microscope. Its magnified view shows the relationships between the Cr film-edge, cross-filament, scan direction, and THz polarization. **b** Normalized resolution curves for various spectral components. **c** Normalized resolution curves for various heights *h* between the cross-filament and the sample surface. **d** Normalized resolution curves for various pulse energies of the control beams

A knife-edge method was used to quantitatively determine the spatial resolution of the THz near-field microscope. The sample was a 1 mm ×1 mm Cr film on a glass substrate, as shown in Fig. 3a, and the polarization of the incident THz beam was along the *X-*direction. A magnified view shows that the Cr film-edge was raster scanned with a 20 µm step along the *X* direction (light-blue arrow). Resolutions of the various THz spectral components were investigated. At each scan point, the THz near-field signal was measured and a Fourier transformation was applied to acquire the THz spectrum. The THz intensity value $\left| {E_{{{{\mathrm{THz}}}}}\left( v \right)} \right|^2$ for each spectral component was calculated to plot the resolution curve. The height *h* and the pulse energies of the control beams were again fixed at 50 µm and 0.3 mJ, respectively. Three normalized resolution curves for 0.94, 1.35, and 1.91 THz were extracted and plotted in Fig. 3b. The higher-frequency spectral components exhibited sharper resolution curves. A 10% to 90% criterion was adopted to estimate the 165 µm, 134 µm, and 81 µm resolutions for the 0.94, 1.35, and 1.91 THz components, respectively, which corresponded to 0.52*λ*_0.94 THz_, 0.60*λ*_1.35 THz_, and 0.52*λ*_1.91 THz_, where *λ* was the wavelength of the corresponding spectral component (see section III-b in the SI). The data indicated that the diffraction effect was more significant and the resolution-to-wavelength ratio was smaller for lower-frequency components and a fixed propagation distance. Note that the ratio at 1.35 THz was higher than that at 1.91 THz. Two factors were responsible for this. On one hand, the dispersive modulation of the THz beam by the cross-filament and the quasi-Gaussian distribution of the plasma density^34–36^ both affected the resolution of the spectral component. On the other hand, the low SNR of the 1.91 THz component may also have led to measurement errors. To further understand the resolution characteristics, we used an “aperture-transmission” simulation model (see section III-a in the SI). The simulation results for 0.94 and 1.35 THz were basically consistent with the experimental data, which indicated that the diffraction effect was the primary factor that determined the resolution (see section III-b in the SI). Meanwhile, differences between the simulation and experiment could be observed, which indicated that the dispersive modulation by the cross-filament and the non-uniform distribution of the plasma density could also have affected the resolution.

Resolutions obtained with various heights *h* of the cross-filament above the sample surface were analyzed. The sample was sequentially adjusted to increase *h*, and the pulse energies of the control beams were 0.3 mJ. For each *h*, the normalized resolution curve for 1.35 THz was extracted as discussed above. Experimental results for *h* = 50, 100, 150, 200, and 250 µm were plotted in Fig. 3c. For larger *h*, the resolution was deteriorated to a greater extent by the diffraction effect. By using the 10–90% criterion, the resolutions for *h* = 50, 100, 150, 200, and 250 µm were estimated to be 134, 182, 208, 224, and 270 µm, respectively. Resolutions for the various *h* were also simulated with the aperture-transmission model. The experimental and simulation results were basically consistent (see section III-c in the SI). Slight deviations were attributed to the experimental alignment error for *h*.

Resolutions obtained with various control beam pulse energies were also examined. By adjusting a tunable neutral density attenuator (A2, see section I-a in the SI), the pulse energies were simultaneously fixed at 0.1, 0.2, 0.3, 0.4, and 0.5 mJ, while *h* was fixed at 50 µm. As above, the normalized resolution curves for different pulse energies were extracted at 1.35 THz and plotted in Fig. 3d. By using the 10% to 90% criterion, the resolutions for 0.1, 0.2, 0.3, 0.4, and 0.5 mJ pulse energies were 136, 130, 134, 105, and 91 µm, respectively. Resolution curves for higher pulse energies should have been smoother because the cross-sections of the cross-filaments were larger. This discrepancy was attributed to the non-uniform distribution of the plasma density, because the density inside a filament is quasi-Gaussian^34–36^, and the overlapped plasmas strengthened the steepness of the density. With increasing pulse energies, the plasma density in the central part of the cross-filament should have been sharper, steepening the resolution curve. The intensity of the modulated THz signal was amplified and the sample surface was more easily damaged with increasing pulse energies (see section III-d in the SI). Therefore, a balance between resolution, SNR, and sample damage had to be carefully considered.

### Application cases

**Fig. 4:**
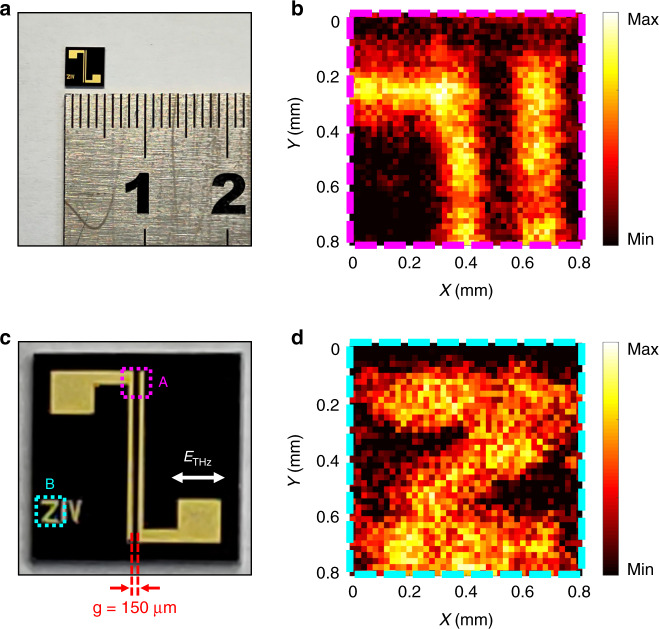
THz near-field imaging of a semiconductor chip. **a** Semiconductor chip and a ruler. The chip was a THz antenna. Its electrode material was an AuGeNi-alloy on a semi-insulating gallium arsenide (SI-GaAs) substrate. **c** Magnified view of the chip. The slit width of the THz antenna was 150 µm, and the THz polarization was perpendicular to the electrodes. Two measurement regions A and B were marked by purple and light-blue dashed boxes. The near-field images of the regions for 1.35 THz are shown in **b** and **d**, respectively

This method of THz near-field microscopy was applicable to many different materials. Three samples were examined: a semiconductor chip, a plastic pattern, and a greasy spot. The semiconductor chip in Fig. 4a, c was a THz antenna with an AuGeNi-alloy electrode fabricated via photolithography on a semi-insulating gallium arsenide (SI-GaAs) substrate^37^. Its slit width was 150 µm and the THz polarization was perpendicular to the electrodes. The sample was raster scanned with a 20 µm step. Two 40 × 40-pixel measurement regions A and B, marked by purple and light-blue dashed boxes, are shown in Fig. 4c. During imaging, *h* was adjusted to approximately 100 µm to ensure adequate resolution without sample damage from the 0.3 mJ control beams. Near-field images of regions A and B for 1.35 THz are shown in Fig. 4b and d, respectively. The THz reflectivity values measured in the metallic regions were higher than that of the SI-GaAs substrate. Fine structures of the sample could be identified, including the THz antenna and the letter “Z”. The experiment thus demonstrated that the technique could be used to characterize delicate morphological information.

**Fig. 5:**
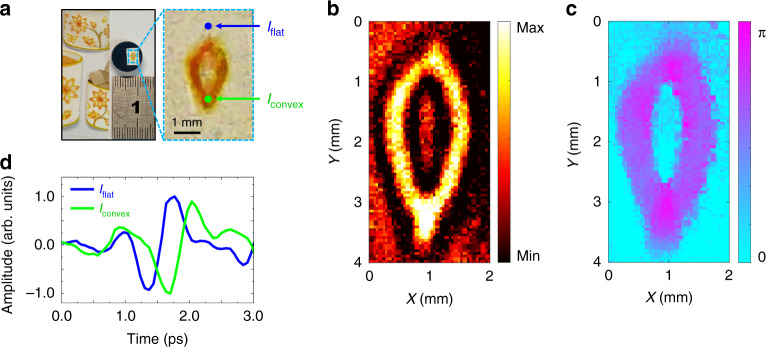
THz near-field imaging of a plastic pattern. **a** Plastic pattern, its petal-shaped region, and a ruler. The magnified view shows the petal shape, which was approximately 1.1 mm × 2.7 mm. Positions of the THz signals *I*_flat_ and *I*_convex_ are marked by blue and green points on the flat and convex regions of the petal. **d** THz temporal *I*_flat_ and *I*_convex_ signals. Intensity and phase images of the petal at 0.7 THz are presented in **b** and **c**, respectively

The intensity and phase of the THz near-field signal were obtained simultaneously and both could be used to analyze sample features. A plastic pattern pasted on a metallic mirror is shown in Fig. 5a. A magnified view shows a 1.1 mm × 2.7 mm petal shape in the pattern. The THz temporal signals *I*_flat_ and *I*_convex_ were measured on the flat and convex regions of the petal, respectively, as marked by blue and green points in Fig. 5a. In Fig. 5d, *I*_convex_ exhibited a significant time delay and waveform distortion relative to *I*_flat_, because the convex region had a longer optical path and a larger THz dispersion. Therefore, the *I*_flat_ and *I*_convex_ differences were easily distinguished. The petal pattern was raster scanned with a 50 µm step, and the measurement region was 40 × 80 pixels. Because the sample was larger in size than the semiconductor chip and had an uneven surface, the height *h* was fixed at 200 µm to prevent sample damage. The pulse energies were fixed at 0.3 mJ to ensure an enough SNR of the THz near-field signal. After Fourier transformations were implemented, the intensity and phase images of the petal pattern acquired at 0.7 THz were selected to present the sample morphology because of their high imaging qualities (see section IV-a in the SI), as shown in Fig. 5b and c, respectively. The petal shape was observed, and the edge of the petal exhibited a stronger intensity and larger phase signal because of a spectral distortion and a time delay induced by the sample. The experiment demonstrated that both THz intensity and phase information could be utilized to characterize the sample, while the sample remained undamaged during imaging.

**Fig. 6:**
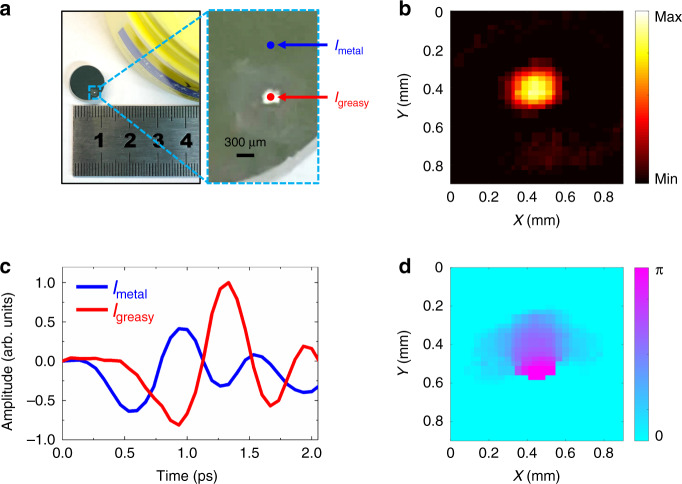
THz near-field imaging of a greasy spot. **a** Greasy spot (Vaseline cream) on a metallic mirror, and a ruler. A magnified view shows the 300 µm diameter of the spot. The positions of the *I*_metal_ and *I*_greasy_ THz signals are marked by blue and red points on regions of the mirror and the spot. **c** THz temporal signals of *I*_metal_ and *I*_greasy_. Intensity and phase images of the spot at 1.0 THz are presented in **b** and **d**, respectively

Soft materials, such as those in biomedical sensing and chemical inspection, can be examined with the THz near-field technique. Here, imaging of a greasy spot (a drop of Vaseline cream) on the surface of a metallic mirror was characterized, as shown in Fig. 6a. The spot diameter was approximately 300 µm. The THz signals *I*_metal_ and *I*_greasy_ were acquired in regions of the mirror and the spot, marked by blue and red points, respectively, in Fig. 6a. Figure 6c shows the *I*_metal_ and *I*_greasy_ temporal signals. Relative to *I*_metal_, *I*_greasy_ had a significant time delay because the refractive index of the Vaseline cream was higher than that of air. The *I*_greasy_ amplitude was higher than that of *I*_metal_, possibly because the quasi-hemispherical spot may have created a lens effect that enhanced the reflective signal of the incident THz beam. Differences between *I*_metal_ and *I*_greasy_ could be easily discerned. The greasy spot was raster scanned with a 30 µm step, the measurement region was 30 × 30 pixels, *h* was 200 µm, and the pulse energies were 0.3 mJ. After Fourier transformations, the intensity and phase images of the greasy spot at 1.0 THz are shown in Fig. 6b and d, respectively. The spot was clearly observed. In addition, the bottom region of the sample had a greater thickness relative to the sample thickness in other regions, and this caused greater phase accumulation in the THz signals. This phenomenon was displayed accurately in the phase image, as shown in Fig. 6d. The experiment thus demonstrated the ability to characterize soft materials.

## Discussion

Relative to previous reports on THz near-field microscopy^5,6,13,14,38,39^, the advantage of our method was not the resolution capability, but the fact that no THz detector or source was close to the sample surface, ensuring that the technique was applicable to different types of samples, such as metals, semiconductors, colloid, and fluidic materials. In the previous reports, it was essential that a THz detector or source approached the sample with a metallic aperture^40^, scattering tip^41^, film photomodulator^38^, spintronic THz emitter^14^, electro-optic crystal^39^, or micro-structured photo-conductive antenna^9^. Soft materials could thus be easily damaged and the THz detector or source could be contaminated. Here, we only needed to adjust the height *h* and the pulse energies of the control beams to prevent sample damage, as demonstrated in the greasy spot imaging. The scheme is also suitable in principle for an encapsulated sample, if its packaging is transparent to THz and visible light. Furthermore, transmission and reflection measurement modes could be simultaneously supported, which expands the testing capability. In summary, it could be anticipated that our method will significantly broaden applications of THz near-field microscopy.

There are several ways for improving the method. In future designs, advanced diffraction optical elements^42,43^ and metasurface devices^44,45^ could be introduced to modulate the wave front of a control beam, and a micro-plasma in three dimensions could be shaped by only a control beam. In this way, the resolution could be further optimized by reducing the size of the plasma. The SNR of the current system was still restricted, which affected the imaging quality. In future improvements, advanced THz emitters could be used, such as organic crystals^46^, lithium niobate^47^, and spintronic emitters^48^. Moreover, advanced digital image processing algorithms could be utilized to more efficiently extract THz near-field information.

In conclusion, a new THz near-field microscopy technique was demonstrated. It was based on an air-plasma dynamic aperture that did not require close approach to a sample by a THz detector or source to obtain quasi sub-wavelength resolution (approximately *λ*/2). Near-field imaging of metallic, semiconductor, plastic, and greasy samples was demonstrated. The resolution characteristics were investigated in detail via the knife-edge method, and an aperture-transmission model was used to examine the physical mechanism. Overall, the method opened a new direction for THz microscopy for fundamental research and industry.

## Materials and methods

Methods and any associated references are available in the Supplementary Information.

## Supplementary information

Supplementary Information for Terahertz Near-field Microscopy based on An Air-Plasma Dynamic Aperture
