# Supplementary material for: Terahertz near-field microscopy based on an air-plasma dynamic aperture
Source: Light Sci Appl. 2022 May 7;11:129. doi: 10.1038/s41377-022-00822-8 (PMC9079089; doi:10.1038/s41377-022-00822-8)
Supplement: Supplementary file 1 — Supplementary Information for Terahertz Near-field Microscopy based on An Air-Plasma Dynamic Aperture [file 41377_2022_822_MOESM1_ESM.docx]

Supplementary Information for Terahertz Near-field Microscopy based on An Air-Plasma Dynamic Aperture

Xin-ke Wang^1^, Jia-sheng Ye^1^, Wen-feng Sun^1^, Peng Han^1^, Lei Hou^2^, and Yan Zhang^1,*^

^1^Beijing Key Laboratory of Metamaterials and Devices, Key Laboratory of Terahertz Optoelectronics Ministry of Education, Department of Physics, Capital Normal University, Beijing, 100048, China.

^2^Applied Physics Department, Xian University of Technology, Xian, Shaanxi, 710048, China.

Correspondence:

Yan Zhang ([yzhang@cnu.edu.cn](mailto:yzhang@cnu.edu.cn))

**I. Experimental details and properties of terahertz (THz) near-field signals**

**I-a. Optical configuration of THz near-field microscopy**


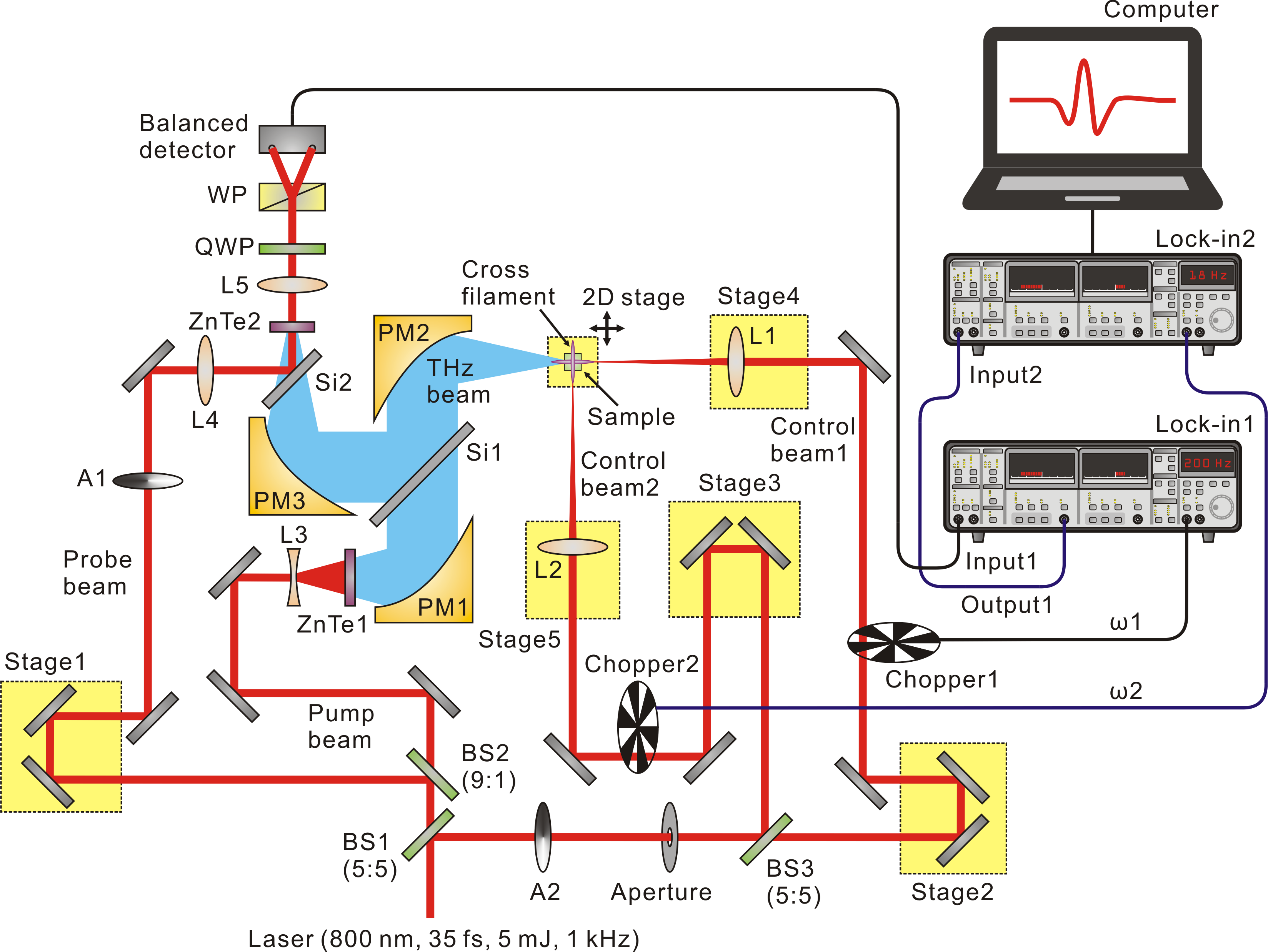


**Figure S1| Optical configuration of the terahertz (THz) near-field microscope based on an air-plasma dynamic aperture.** BS1, BS2, and BS3: non-polarizing beam splitters; A1 and A2: tunable neutral density attenuators; L1, L2, L4, and L5: convex lenses; L3: concave lens; PM1, PM2, and PM3: off-axis parabolic mirrors; Si1 and Si2: high-resistance silicon wafers; QWP: quarter wave plate; WP: Wollaston prism.

The optical configuration of the near-field system is shown in Fig. S1. A 800 nm Ti:sapphire amplifier laser (Spectra-Physics Spitfire Ace-35F) with a 35 fs pulse duration, a 1 kHz repetition rate, and a maximum energy of 5 mJ per pulse was the optical source. The laser pulse was divided by three non-polarizing beam splitters (BS1, BS2, and BS3), into a pump beam, probe beam, Control beam1, and Control beam2. The transmission/reflection ratios of BS1, BS2, and BS3 were 5:5, 9:1, and 5:5, respectively. The pump beam was expanded by a concave lens (L3) with a 5 cm focal length and guided to a zinc telluride crystal (ZnTe1) with a <110> orientation. A THz pulse was emitted via optical rectification [1]. Two off-axis parabolic mirrors (PM1 and PM2) with focal lengths of 10 cm and 15 cm, respectively, were used to collimate and focus the THz beam on the sample surface. Reflected THz signals were guided with a high-resistance silicon wafer (Si1) and focused on another <110>-orientated zinc telluride crystal (ZnTe2) by an off-axis parabolic mirror (PM3) with a 10 cm focal length. After passing through a convex lens (L4), the probe beam was collinear with the THz beam in the ZnTe2 by using another high-resistance silicon wafer (Si2). Then, the probe polarization was modulated by the THz electrical field, and the THz signal was extracted with an electro-optic sampling module consisting of a convex lens (L5), a quarter wave plate (QWP), a Wollaston prism (WP), and a balanced detector [2]. In the probe beam path, a motorized linear stage (Stage1) was used to vary the time delay between the pump and probe beams to obtain a THz temporal signal. A tunable neutral density attenuator (A1) was used to limit the power of the probe beam.

Control beam1 and Control beam2 were focused by two 15 cm focal length convex lenses (L1 and L2) to generate two air-plasmas that overlapped in mutually perpendicular directions to form a cross-filament. The central part of the cross-filament was aligned with the incident THz beam to create a dynamic aperture for the THz beam. Two motorized linear stages (Stage2 and Stage3) controlled relative time delays of the two air-plasmas. Two other motorized linear stages (Stage4 and Stage5) were mounted with L1 and L2 to control the relative positions of the air-plasmas. A tunable neutral density attenuator (A2) was used to simultaneously control the intensities of the two air-plasmas. In addition, a tunable aperture was used to restrict the diameters of the two control beams to 7 mm. Samples were fixed on a two-dimensional (2D) motorized stage for scanned imaging, and the sample distance from the cross-filament was carefully adjusted for acquiring near-field images.

A double modulation scheme with two lock-in amplifiers (Stanford Research Systems SR830) was used to acquire THz near-field signals modulated by the central part of the cross-filament [3]. Two mechanical choppers (Chopper1 and Chopper2) were inserted in the paths of Control beam1 and Control beam2, and their frequencies were separately set at *ω*_1_=200 Hz and *ω*_2_=18 Hz, respectively. The signal from the balanced detector was sent to the first lock-in amplifier using 200 Hz as a reference frequency. Its output was sent to another lock-in amplifier with an 18 Hz reference frequency. In this way, the THz near-field signal modulated by the central part of the cross-filament was extracted and the background signal modulated by other parts of the cross-filament was filtered out.

**I-b. THz signals modulated by each single air-plasma**


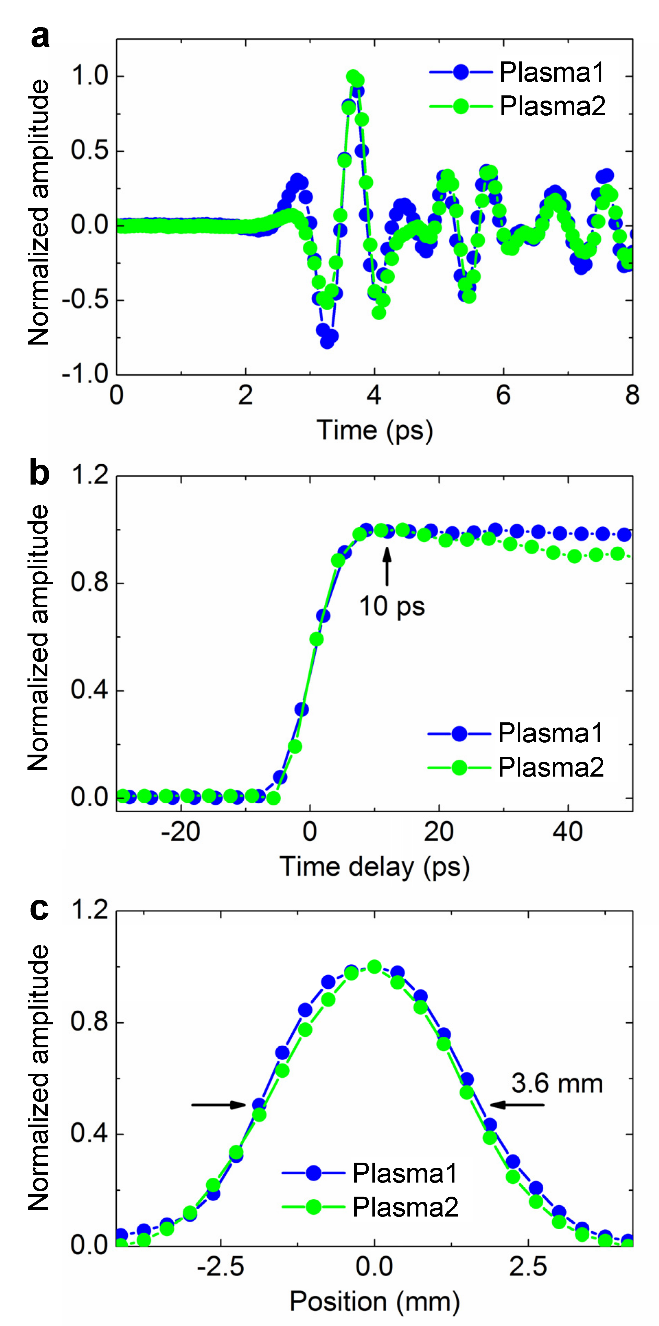


**Figure S2| THz signals modulated by each single air-plasma. a,** Normalized THz temporal signals modulated by each single air-plasma. **b,** Variations in the THz peak signals with time delays between the THz and control beams. **c,** Variations in the THz peak signals with the relative positions of each single air-plasma.

To optimize the THz near-field signal, the THz signals modulated by each single air-plasma were monitored and tuned. A metallic plate was used as a reference sample. The normalized reflected THz temporal signals modulated by each single air-plasma were acquired by using a single modulation scheme and one lock-in amplifier, as shown in Fig. S2a. In the measurement, the other air-plasma was blocked. Both THz signals had identical polarities and similar evolution tendencies in the time domain, which indicated that the modulation effects of the two air-plasmas were analogous. To observe the time-resolved properties of these modulation effects, variations in the two THz signals were obtained by scanning Stage2 and Stage3, while fixing the relative time delay between the THz and probe beams at the THz temporal peak position, as shown in Fig. S2b. Negative time delays indicated that a control beam arrived at the sample after the THz beam, which prevented the THz signal from being modulated by the air-plasma. Both modulated THz signals had smooth variation trends over tens of picoseconds. The time delays between the THz and two control beams were 10 ps to ensure that the entire THz signal was adequately modulated by an air-plasma in the time domain. The relative positions of the two air-plasmas vs. the THz beam were adjusted by scanning Stage4 and Stage5; variations in the THz peak signals are shown in Fig. S2c. Both curves exhibited Gaussian features with a 3.6 mm full-width at half-maximum (FWHM) that was simultaneously dependent on the length of the air-plasma and the diameter of the THz focal spot. The positions of the two maximum THz signals were aligned by adjusting Stage4 and Stage5 to ensure that the THz near-field signal was adequately modulated in the spatial domain. In that case, the central regions of the two air-plasmas overlapped.

**I-c. THz near-field signal modulated by the cross-filament**


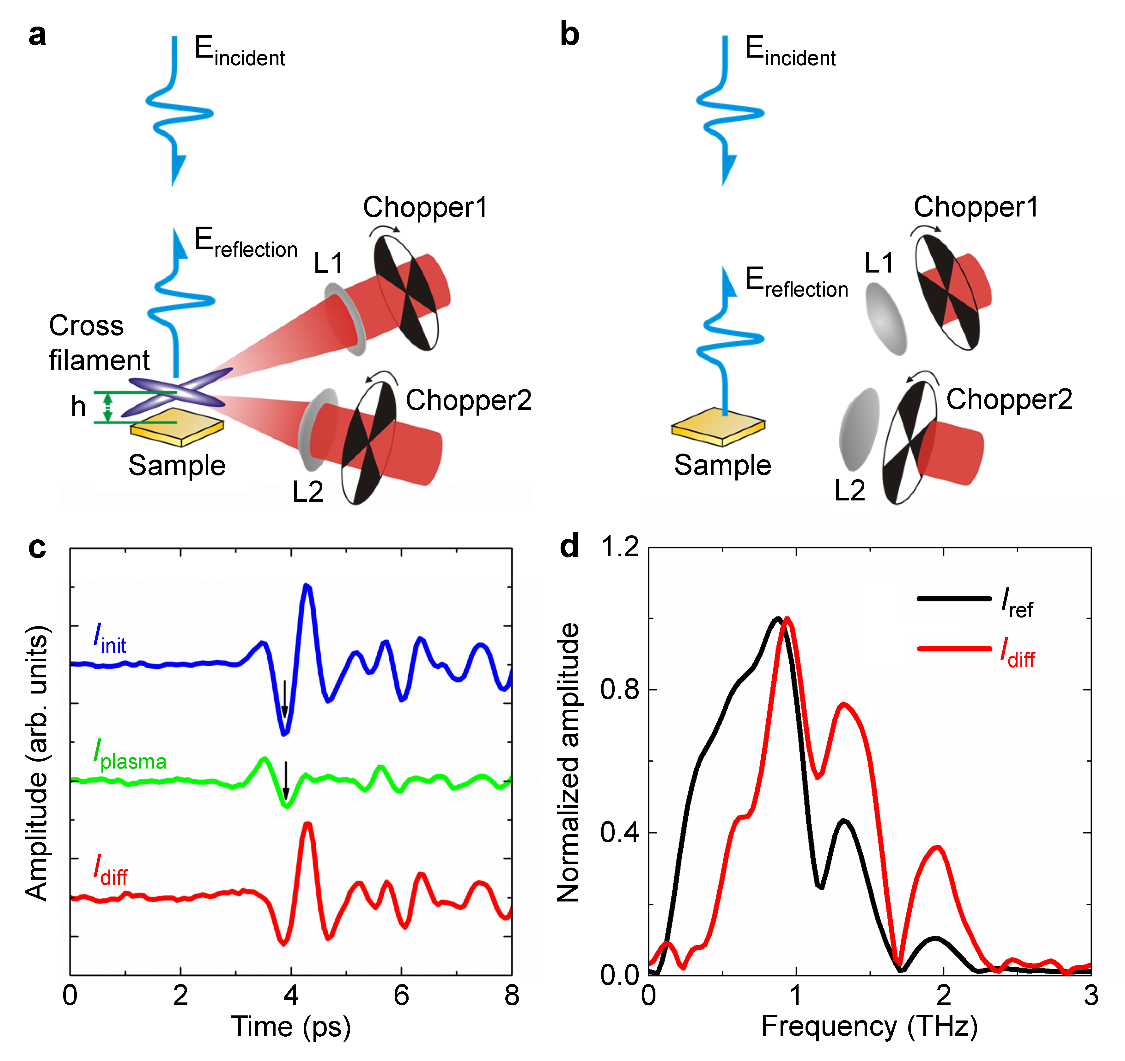


**Figure S3****| Properties of a THz near-field signal modulated by the cross-filament. a and b,** Schematics of THz signals reflected by the cross-filament and sample. **c,** Initial THz near-field signal *I*_init_, THz signal *I*_plasma_ reflected by the cross-filament, and their difference *I*_diff_. **d,** Normalized reference (*I*_ref_) and near-field THz spectra.

When a THz near-field signal modulated by the cross-filament was acquired, it contained an interference between the two THz signals reflected by the sample and the cross-filament. Figures S3a and S3b show schematics of the two THz signals, respectively. When the two choppers allowed the control beams to pass through, the cross-filament was formed that reflected the incident THz beam. When they were blocked by the choppers, the THz beam irradiated the sample surface and was reflected. By using lock-ins, both reflected THz signals were acquired at the same time, so the effect of the THz signal reflected by the cross-filament should be filtered from the final result. Figure S3c shows the processing procedure. A metallic plate was used as a reference sample to reflect the THz signal. An initial THz signal *I*_init_ was obtained, which included the two reflected THz signals from the cross-filament and sample. Then, the metallic plate was withdrawn and the THz signal *I*_plasma_ reflected by the cross-filament was exclusively acquired. Their difference then yielded the final THz near-field signal *I*_diff_. In Fig. S3c, two black arrows indicated that the *I*_plasma_ peak position and the relative position of *I*_plasma_ in *I*_init_. In addition, *I*_plasma_ could be used as a criterion to estimate the height *h* between the cross-filament and the sample surface. To observe the spectral modulation effect of the cross-filament, Fourier transformations were separately performed on the reference THz signal *I*_ref_ (see Fig. 1b) and *I*_diff_. Normalized spectra of *I*_ref_ and *I*_diff_ were plotted in Fig. S3d. The central frequency of *I*_ref_ was approximately 0.9 THz and the spectrum of *I*_ref_ had two dips at 1.17 THz and 1.70 THz because of water vapor absorption and dispersion [4]. In the *I*_diff_ spectrum, the two dips remained, but the central frequency moved to 1.35 THz. This indicated that the modulations of various THz spectral components by the cross-filament were diverse. According to Ref. [5], the plasma characteristics were dispersive in the THz frequency range, which corresponded to our results well.

**II Details for THz imaging**

**II-a. Optical configuration of THz far-field imaging**


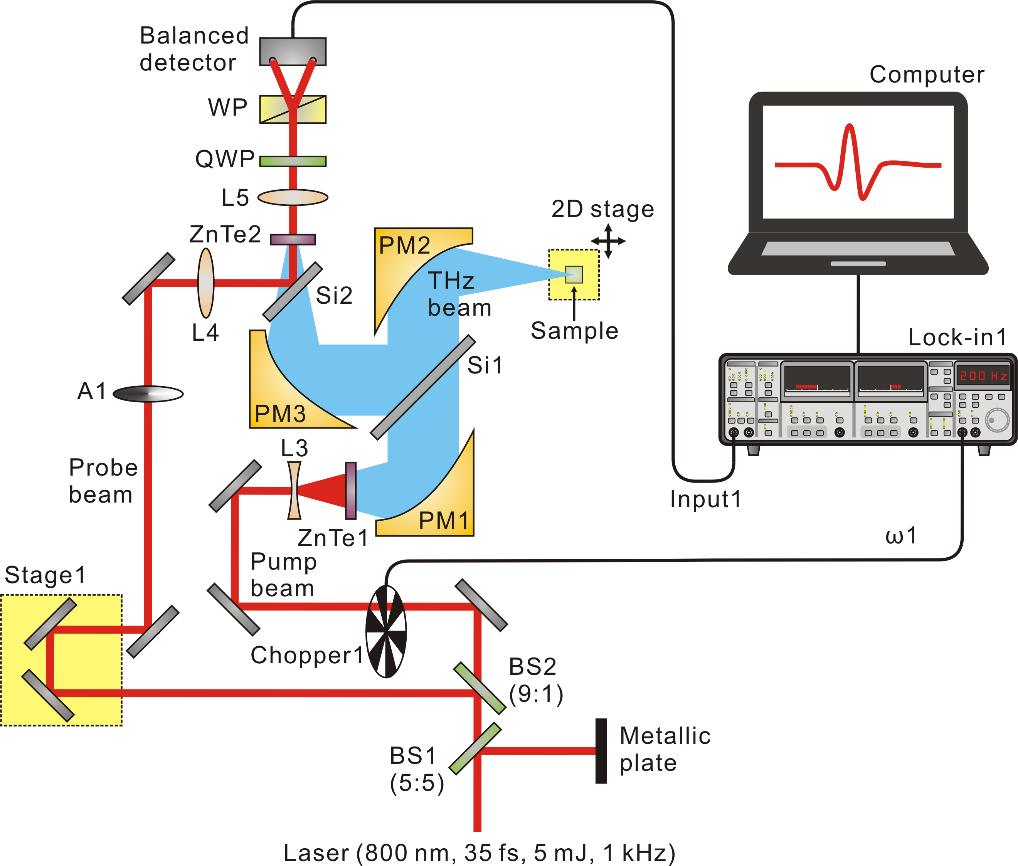


**Figure S4|** **Optical configuration of the THz far-field imaging system.**

The optical configuration of the THz far-field imaging system is shown in Fig. S4 and is similar to that of the near-field system, with the exception of the part of the system for the control beams. A metallic plate was used to block the control beams. Chopper1 with *ω*_1_=200 Hz was inserted in the path of the pump beam, and its output was sent to the lock-in amplifier to act as the reference frequency. The signal from the balanced detector was input to the lock-in amplifier and was then read using a computer.

**II-b. Resolution estimation of far-field THz imaging**


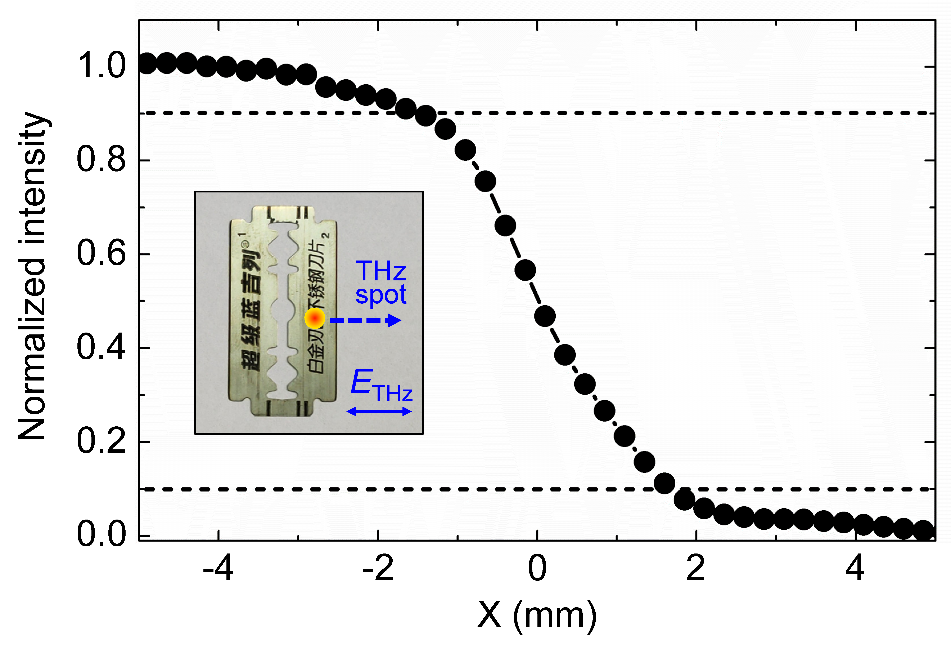


**Figure S5| Resolution of far-field THz imaging via the knife-edge method.** A razor with a 100 μm thickness was mounted at the focal spot of the incident THz beam and its edge was raster scanned with a 300 μm step along the *X* direction. At each scan point, the THz spectral intensity $\left|E_{\text{THz}}\left(v\right)\right|^{2}$ was extracted and the normalized resolution curve for 1.35 THz was plotted, where $E_{\text{THz}}\left(v\right)$ is the THz spectral amplitude and $v$ is the frequency. The inset shows the relationships of the razor-edge, the THz focal spot, the scan direction, and the THz polarization.

A knife-edge method was used to estimate the resolution of THz far-field imaging. A razor with a 100 μm thickness was the sample, as shown in the inset of Fig. S5. The sample was mounted at the focal spot of the incident THz beam, and the THz polarization was perpendicular to the razor’s edge. The edge was raster scanned with a 300 μm step along the *X* direction (see the blue arrow in the inset) and the reflected far-field THz signal was detected. At each scan point, the THz spectral intensity $\left|E_{\text{THz}}\left(v\right)\right|^{2}$ was extracted by implementing a Fourier transformation and the normalized resolution curve for 1.35 THz was plotted in Fig. S5. Here, $E_{\text{THz}}\left(v\right)$ is the THz spectral amplitude and $v$ is the frequency. We used the 10% to 90% criterion to estimate the spatial resolution, which was approximately 3.2 mm. The result illustrates that the resolution was greatly improved by the near-field method.

**II-c. Measurement results of three resolution test charts**


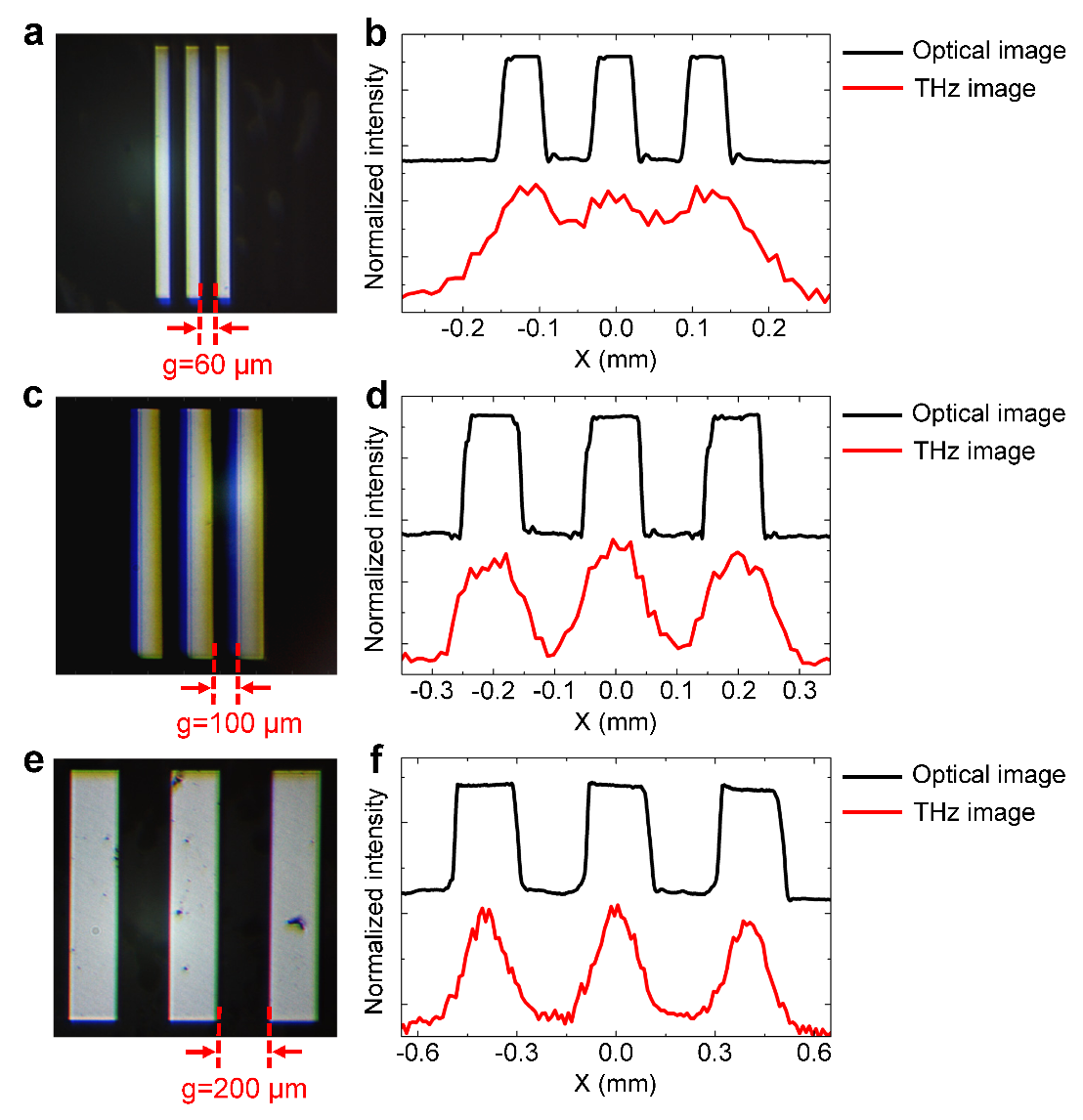


**Figure S6| Measurements of three resolution test charts. a, c, e,** Optical micrographs of three resolution test charts with slit widths of 60 μm, 100 μm, and 200 μm. **b, d, f,** Normalized 1.35 THz and optical intensity profiles of the charts, obtained with THz near-field microscopy and an optical microscope.

Three resolution test charts with slit widths of 60 µm, 100 µm, and 200 µm were imaged with THz near-field microscopy. The normalized intensity profiles for 1.35 THz were extracted and plotted in Figs. S6b, S6d, and S6f, respectively. Micrographs were acquired with an optical microscope, as shown in Figs. S6a, S6c, and S6e, respectively. The optical and THz near-field measurements were completely consistent.

**III Aperture-transmission model and resolution estimations**

**III-a. Model details**


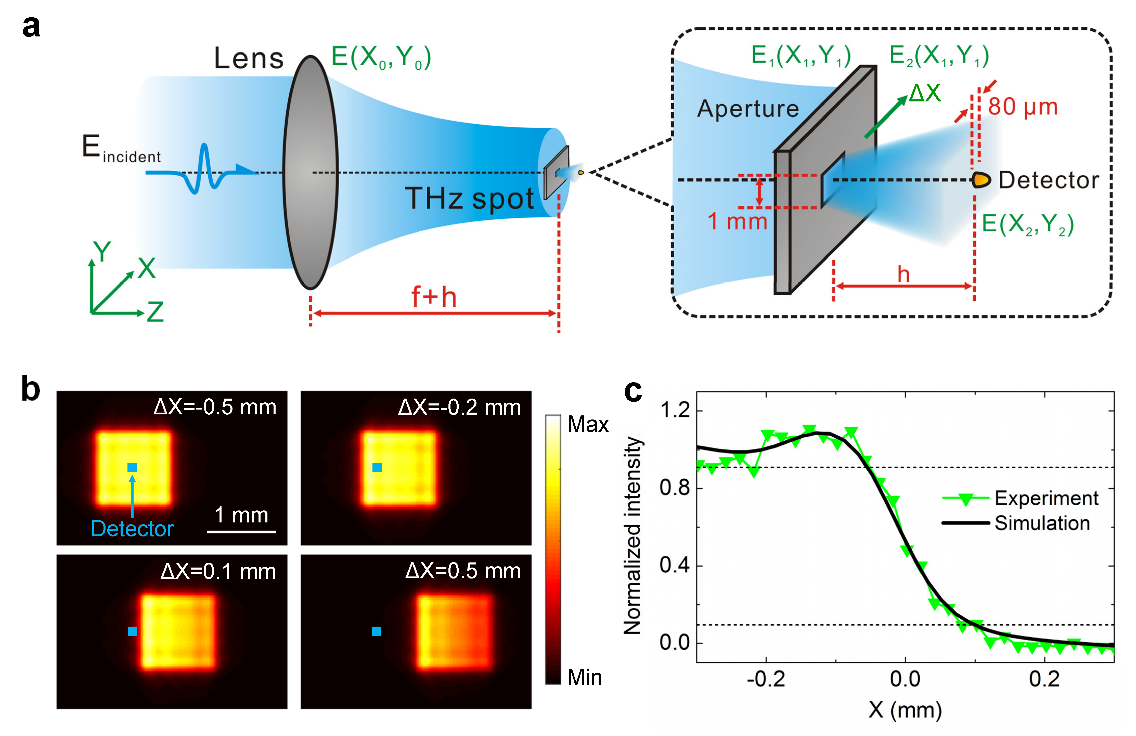


**Figure S7| Aperture-transmission model and simulation results. a,** Schematic of the “aperture-transmission” model. The incident THz beam was focused by a convex lens with a focal length of *f*=15 cm. A 1 mm×1 mm aperture was set at the focal spot. The transmitted THz beam from the aperture was monitored with a square detector with a side length of 80 µm. *f+h* was the distance between the lens and the aperture. *h* was the distance between the aperture and the detector. *E*(*X*_0_, *Y*_0_), *E*_1_(*X*_1_, *Y*_1_), *E*_2_(*X*_1_, *Y*_1_), and *E*(*X*_2_, *Y*_2_) were THz fields at the exit face of the lens, the entry and exit faces of the aperture, and the entry face of the detector, respectively. The aperture position was adjusted along the *X* direction. Δ*X* was the moving distance. At each scan point, the averaged THz intensity was acquired to shape the simulated resolution curve. **b,** Simulated THz amplitude distributions for the 1.35 THz component at the entry face of the detector for Δ*X*=-0.5 mm, -0.2 mm, 0.1 mm, and 0.5 mm. *h* was fixed at 50 µm, and the detector was marked by a light-blue square. **c,** Normalized simulated and experimental resolution curves at 1.35 THz.

To understand the resolution characteristics of the THz near-field microscope, an “aperture-transmission” model was developed, as schematically depicted in Fig. S7a. The incident THz beam was focused by a convex lens with a focal length of *f*=15 cm (corresponding to the focal length of PM2). At the focal spot, a 1 mm×1 mm aperture was set that corresponded to the size of the Cr film. The transmitted THz beam from the aperture was monitored with a square detector with a side length of 80 µm, corresponding to the central part of the cross-filament. When the height *h* between the cross-filament and the sample was adjusted, the distance between the sample and PM2 was varied. Therefore, the distance between the lens and the aperture was *f+h*. The distance between the aperture and the detector was *h*. *E*(*X*_0_, *Y*_0_) was the THz field at the exit face of the lens. *E*_1_(*X*_1_, *Y*_1_) and *E*_2_(*X* _1_, *Y* _1_) were THz fields at the entry and exit faces of the aperture. The thickness of the aperture was neglected. *E*(*X* _2_, *Y* _2_) was the THz field at the entry face of the detector. (*X* _0_, *Y* _0_), (*X* _1_, *Y* _1_), and (*X* _2_, *Y* _2_) were spatial coordinates on the corresponding planes. The propagation of the THz beam between the lens and the aperture was described by Fresnel diffraction [6]. *E*(*X* _0_, *Y* _0_) and *E*_1_(*X* _1_, *Y* _1_) could be expressed as

$E\left(X_{0},Y_{0}\right)=\exp\left[-\frac{\left(X_{0}^{2}+Y_{0}^{2}\right)}{W^{2}}\right]\exp\left[-j\frac{k}{2f}\left(X_{0}^{2}+Y_{0}^{2}\right)\right]$ (1)

$E_{1}\left(X_{1},Y_{1}\right)=\frac{1}{j\lambda\left(f+h\right)}\iint E\left(X_{0},Y_{0}\right)\exp\left\{j\frac{k}{2\left(f+h\right)}\left[\left(X_{0}-X_{1}\right)^{2}+\left(Y_{0}-Y_{1}\right)^{2}\right]\right\}dX_{0}dY_{0}$(2)

where *k* is the wave-number in vacuum, *λ* is the wavelength of the incident THz beam, *W* is the 6 mm radius of the incident THz beam. The relationship between *E*_1_(*X*_1_, *Y*_1_) and *E*_2_(*X*_1_, *Y*_1_) is given by

$E_{2}\left(X_{1},Y_{1}\right)=E_{1}\left(X_{1},Y_{1}\right)\cdot T\left(X_{1},Y_{1}\right)$ (3)

$T\left(X_{1},Y_{1}\right)=\left\{\begin{matrix} 0\text{ Outside of the aperture} \\ 1\text{ Inside of the aperture} \end{matrix}\right.$ (4)

where *T*(*X* _1_, *Y* _1_) is the transmission function of the aperture. Because the propagation distance between the aperture and the detector was smaller than the wavelength of the incident THz beam, a near-field scalar diffraction algorithm was used to fulfill Rayleigh-Sommerfeld diffraction [7]. *E*(*X* _2_, *Y* _2_) could be written as

$E\left(X_{2},Y_{2}\right)=\frac{kh}{j2\pi}\iint E_{2}\left(X_{1},Y_{1}\right)\frac{\exp\left(jk\rho\right)}{\rho^{2}}\left(1-\frac{1}{jk\rho}\right)dX_{1}dY_{1}$ (5)

where $\rho=\sqrt{\left(X_{2}-X_{1}\right)^{2}+\left(Y_{2}-Y_{1}\right)^{2}+h^{2}}$. In the simulation, the position of the aperture was adjusted along the *X* direction in sequence and Δ*X* was the moving distance. At each scan point, THz amplitudes were averaged in the detection region and a THz intensity value $\left|E_{\text{THz}}\left(v\right)\right|^{2}$ was obtained for the resolution curve.

To verify the model, a case with *h=*50 µm and *v=*1.35 THz was simulated. The moving step of the aperture was fixed at 20 μm. The THz amplitude distributions for Δ*X*=-0.5 mm, -0.2 mm, 0.1 mm, and 0.5 mm were given in Fig. S7b at the entry face of the detector. When Δ*X*=-0.5 mm, the detector and the THz focal spot were collinear with the center of the aperture. The position of the detector was marked by a light-blue square. When varying the position of the aperture, the transmitted THz intensity was reduced because the aperture deviated from the THz focal spot. Figure S7c shows the normalized simulated and experimental resolution curves for 1.35 THz, which agree well. This demonstrated that the diffraction effect was the primary factor that determined the resolution of the THz near-field microscope.

**III-b. Details on resolutions for various spectral components**

Figure S8a shows the THz intensity distribution of the Cr film on a glass substrate for various frequencies. The white dashed line was used to separate the glass region from the Cr film. The THz intensity from the Cr film was stronger than that from the glass substrate because of its higher reflectivity. In addition, the THz spectrum had two dips at 1.17 THz and 1.70 THz that were consistent with the experimental results shown in Fig. S3d. By using the aperture-transmission model, the normalized resolution curves at 0.94 THz, 1.35 THz, and 1.91 THz were simulated and plotted in Fig. S8b. In the simulation, *h* was fixed at 50 µm, and the moving step of the aperture was 20 µm. The simulated results were similar to the experimental results in Fig. 3b, where the higher-frequency spectral components had sharper resolution curves. The 10% to 90% criterion was used to evaluate the resolutions. Figure S8c shows the simulated resolutions for 0.94 THz, 1.35 THz, and 1.91 THz, which were 186 µm, 152 µm, and 136 µm, respectively. The experimental results were also given. The simulated and experimental results at 0.94 THz and 1.35 THz basically agreed, which indicated that the resolution of the THz near-field microscope was mainly determined by the diffraction effect. A deviation between the simulated and experimental results at 1.91 THz was also observed. In the model, the dynamic aperture formed by the cross-filament was the same for all spectral components. The dispersive modulation of the THz beam by the cross-filament and the quasi-Gaussian distribution of the plasma density indicated that the assumption may be limited.


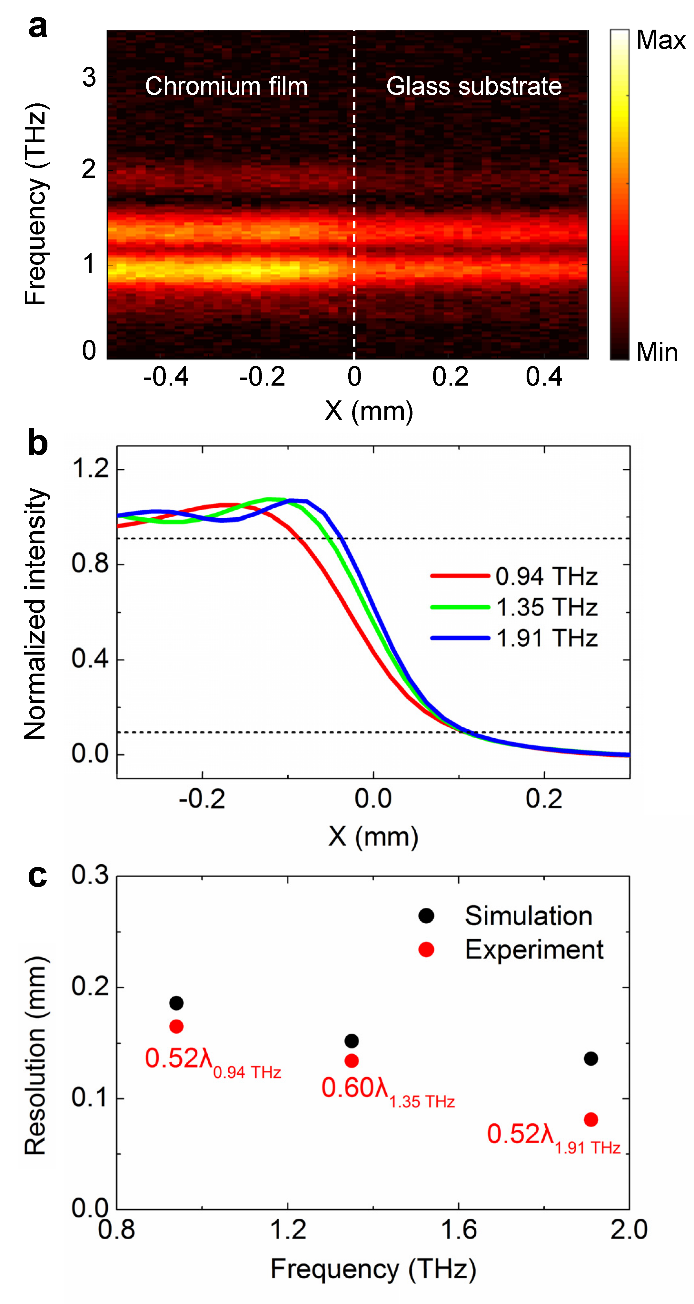


**Figure S8| Resolutions with various spectral components. a,** THz intensity distribution of the Cr film on the glass substrate for various frequencies. The Cr and glass regions were separated by a white dashed line. **b,** Normalized simulated resolution curves for 0.94 THz, 1.35 THz, and 1.91 THz. **c,** Resolutions for 0.94 THz, 1.35 THz, and 1.91 THz from the simulations and experiment.

**III-c. Details on resolutions for various heights between the cross-filament and sample**


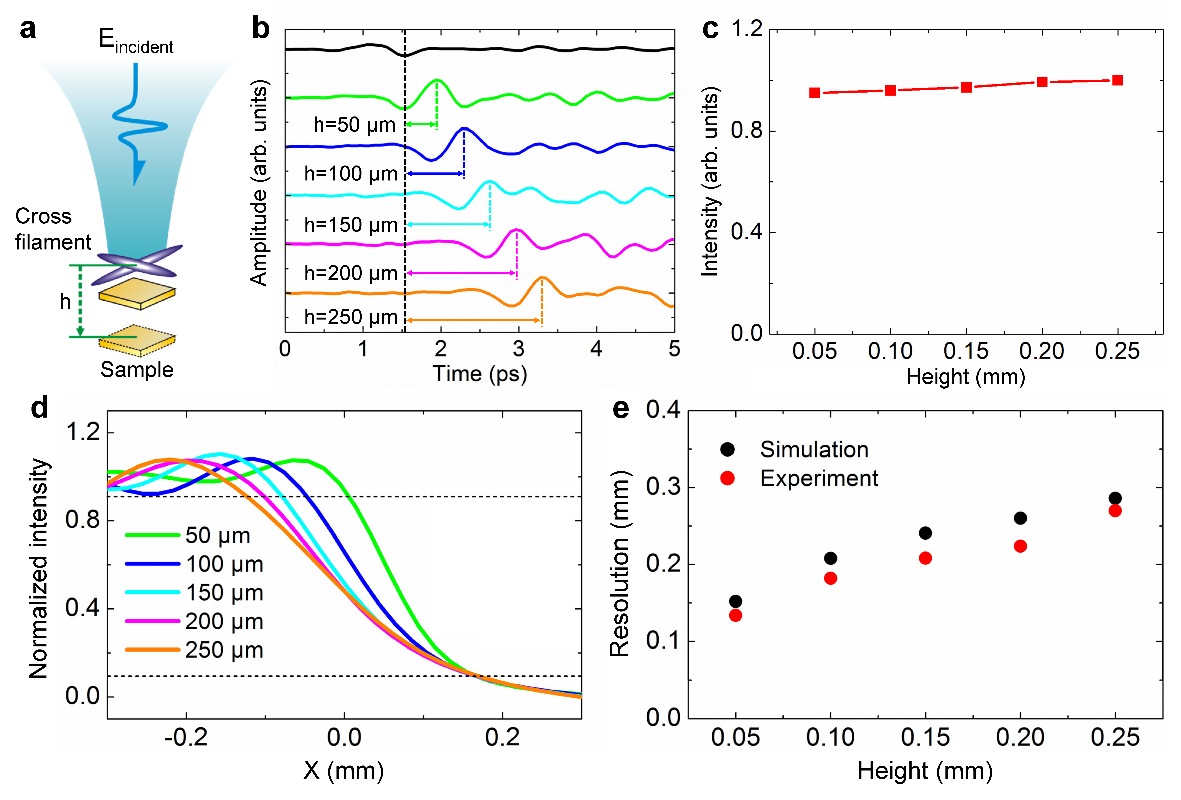


**Figure S9| Resolutions for various heights between the cross-filament and the sample surface. a,** Schematic of adjusting the height *h*. **b,** THz temporal signals reflected by the cross-filament and the Cr film for *h=*50 µm, 100 µm, 150 µm, 200 µm, and 250 µm. **c,** Total THz intensity as a function of *h*. **d,** Normalized simulated resolution curves for *h=*50 µm, 100 µm, 150 µm, 200 µm, and 250 µm. **e,** Resolutions for various *h* in the simulations and experiment.

To check the effects of *h* on the resolution, the sample distance from the cross-filament was adjusted, depicted in Fig. S9a. The difference in position between the THz temporal peaks reflected by the cross-filament and the Cr film was used as the criterion to evaluate *h*. The alignment precision of *h* was approximately 10 µm. The THz temporal signals reflected by the cross-filament and the Cr film for *h=*50 µm, 100 µm, 150 µm, 200 µm, and 250 µm are shown in Fig. S9b. The signals exhibited different evolutions in the time domain, which indicated that the diffraction effect and the dispersive modulation of the cross-filament both led to various temporal waveforms of the THz near-field signals. Therefore, variations of the THz spectra for different *h* were nonlinear. Normalized integral values $\int\left|E_{\text{THz}}\left(v\right)\right|^{2}dv$ of their spectral intensities were calculated and plotted in Fig. S9c. The total intensities of the THz near-field signals had smooth variations with *h*. By using the aperture-transmission model, the normalized simulated resolution curves for *h=*50 µm, 100 µm, 150 µm, 200 µm, and 250 µm were obtained and plotted in Fig. S9d, which were consistent with the experimental results (see Fig. 3c). The simulated resolutions for *h=*50 µm, 100 µm, 150 µm, 200 µm, and 250 µm separately were 152 µm, 208 µm, 240 µm, 260 µm, and 286 µm, respectively, as shown in Fig. S9e, along with the experimental results. The simulated and experimental results basically agreed and the slight discrepancies were attributed to the experimental alignment error of *h*.

**III-d. Details of resolutions with various control beam pulse energies**

The effect of control beam pulse energy on the resolution was examined. A2 was adjusted in sequence and the pulse energies were 0.1 mJ, 0.2 mJ, 0.3 mJ, 0.4 mJ, and 0.5 mJ, respectively. According to Refs. [8-10], these energies were much less than the condition needed for multiple filamentation, and plasma movement from Kerr self-focusing was not significant. We aligned L1 and L2 to ensure that the central parts of the two air-plasmas always overlapped. Figure S10a shows the THz spectra for various pulse energies, which had identical profiles and a linear increasing trend. Their total intensities were obtained by calculating $\int\left|E_{\text{THz}}\left(v\right)\right|^{2}dv$ and were plotted as a function of a pulse energy, as shown in Fig. S10b. Insets show the cross-filaments with pulse energies of 0.1 mJ, 0.2 mJ, 0.3 mJ, 0.4 mJ, and 0.5 mJ. Both the cross-filament and THz intensities were significantly enlarged with increasing the pulse energy. A modulation saturation effect was not observed, which indicated that air in the central region of the cross-filament was not completely ionized [11]. According to Refs. [8,9,12], the plasma volume and density were simultaneously enhanced when the pulse energy increased, which led to increased modulation of the THz intensity. The resolutions for various pulse energies are shown in Fig. S10c. Those for 0.1 mJ, 0.2 mJ, and 0.3 mJ pulse energies were 136 µm, 130 µm, 134 µm, respectively. The resolutions for 0.4 mJ and 0.5 mJ pulse energies were 105 µm and 91 µm, respectively, and attributed to the quasi-Gaussian plasma density in the filaments. With increasing the pulse energy, the steepness of the plasma density progressively strengthened in the superposition region of the two air-plasmas, which improved the resolution.


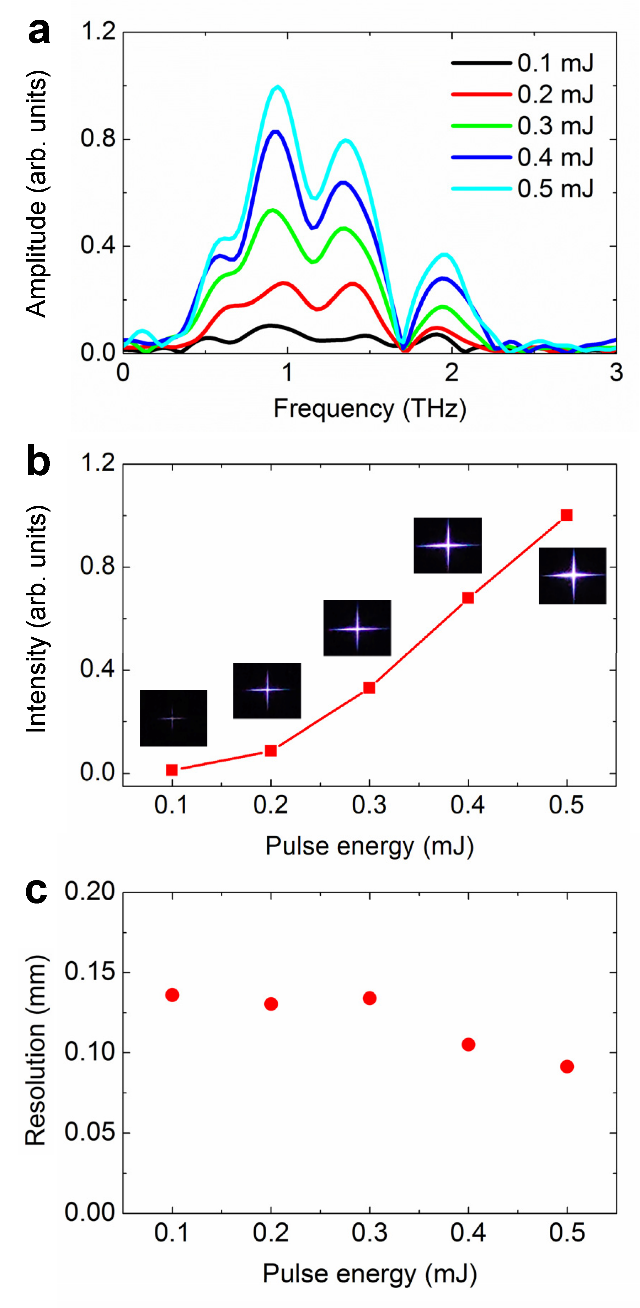


**Figure S10| Resolutions for various control beam pulse energies. a,** THz spectra for pulse energies of 0.1 mJ, 0.2 mJ, 0.3 mJ, 0.4 mJ, and 0.5 mJ. **b,** Total THz intensity as a function of the pulse energy. The insets are cross-filaments with various pulse energies. **c,** Resolutions for various pulse energies.

**III-e. Effect of THz polarization on resolution**


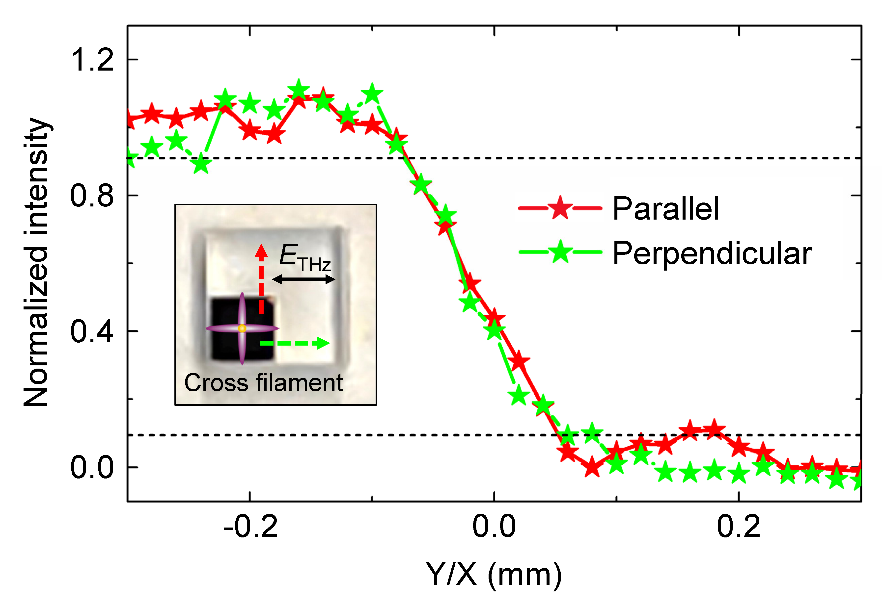


**Figure S11| Resolutions for various THz polarizations.** Cr film-edges were scanned along the *Y* and *X* directions (orientations of the red and green arrows). The inset shows the relationships between the Cr film-edges, the cross-filament, the THz polarization, and the scan directions. The normalized resolution curves for parallel (red line) and perpendicular (green line) polarizations at 1.35 THz were extracted as described above.

Effects of THz polarization on resolution were also checked. The horizontal and vertical edges of the Cr film were separately scanned along the *Y* and *X* directions (red and green arrows in the inset of Fig. S11). The THz polarization was perpendicular to the vertical edge of the Cr film. The inset of Fig. S11 shows the relationships between the Cr film-edges, the cross-filament, the THz polarization, and the scan directions. The pulse energies of the control beams were 0.3 mJ, and *h* was 50 µm. Using the same measurement scheme described above, the normalized resolution curves for parallel (red line) and perpendicular (blue line) polarizations at 1.35 THz were obtained and plotted in Fig. S11. The resolutions were similar at 128 µm and 134 µm for the parallel and perpendicular polarizations, respectively, which was inconsistent with previous reports [13,14]. In general, because of polarization boundary conditions [13], the resolution of a metallic edge with a perpendicular polarization should be smaller than that with a parallel polarization. The reason is still unclear, and needs to be investigated further.

**III-f. Effect of the polarization of the optical filament on resolution**

**
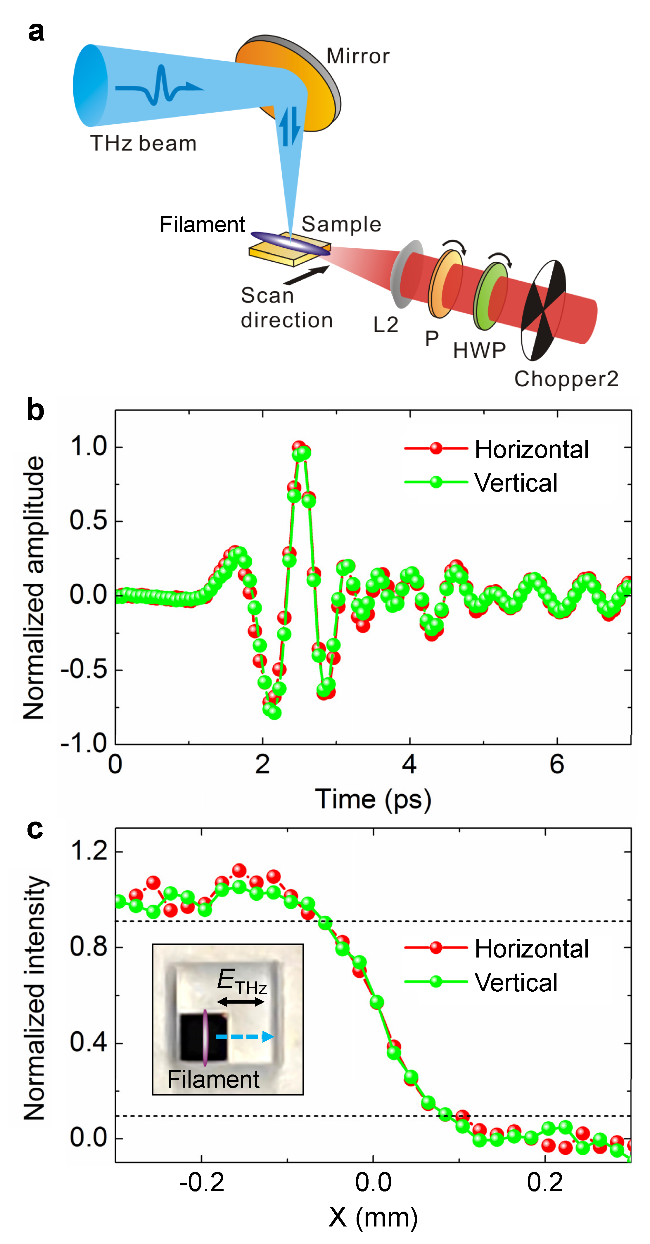
**

**Figure S12| Effect of the polarization of the optical filament on the imaging resolution.** Schematic of a THz signal modulated by an optical filament. The polarization of the filament was adjusted by using a polarizer (P) and a half wave plate (HWP). b, Normalized THz temporal signals modulated by filaments with horizontal and vertical polarizations. c, Normalized resolution curves at 1.35 THz for horizontally and vertically polarized filaments. The inset shows the relationships between the Cr film-edge, the filament, the scan direction, and the THz polarization.

The effect of the polarization of an optical filament on the experimental outcome was also checked. The optical configuration of the experiment is shown in Fig. S12a. A control beam was focused by a lens L2 to form an optical filament. A polarizer (P) and a half wave plate (HWP) were inserted in the path of the control beam and appropriately adjusted to change the polarization of the filament. Chopper2 was used to modulate the output of the filament and its frequency was set at *ω*_2_=200 Hz. The incident THz beam was modulated by the filament and the reflected signal was measured by using a single modulation scheme and one lock-in amplifier. The sample was the Cr film. The THz polarization was perpendicular to the Cr film-edge, as shown in the inset of Fig. S12c. The pulse energy of the control beam was 0.3 mJ and the height *h* was 50 μm. The polarization of the filament was adjusted to a horizontal or vertical direction. Figure S12b shows the normalized THz temporal signals modulated by the horizontally and vertically polarized filaments, which present the same evolution. The Cr film-edge was raster scanned with a 20 μm step along the *X* direction (the light-blue arrow in the inset of Fig. S12c). At each scan point, the THz intensity value $\left|E_{\text{THz}}\left(v\right)\right|^{2}$ for 1.35 THz was obtained to plot the resolution curve. Figure S12c presents the normalized resolution curves for horizontally and vertically polarized filaments, which show the resolutions of 137 μm and 133 μm, respectively. These experimental results manifest that the polarization of the filament has not a significant influence on the outcome.

**IV THz intensity and phase images for different frequencies**

**IV-a. THz intensity and phase images of a plastic pattern**

**
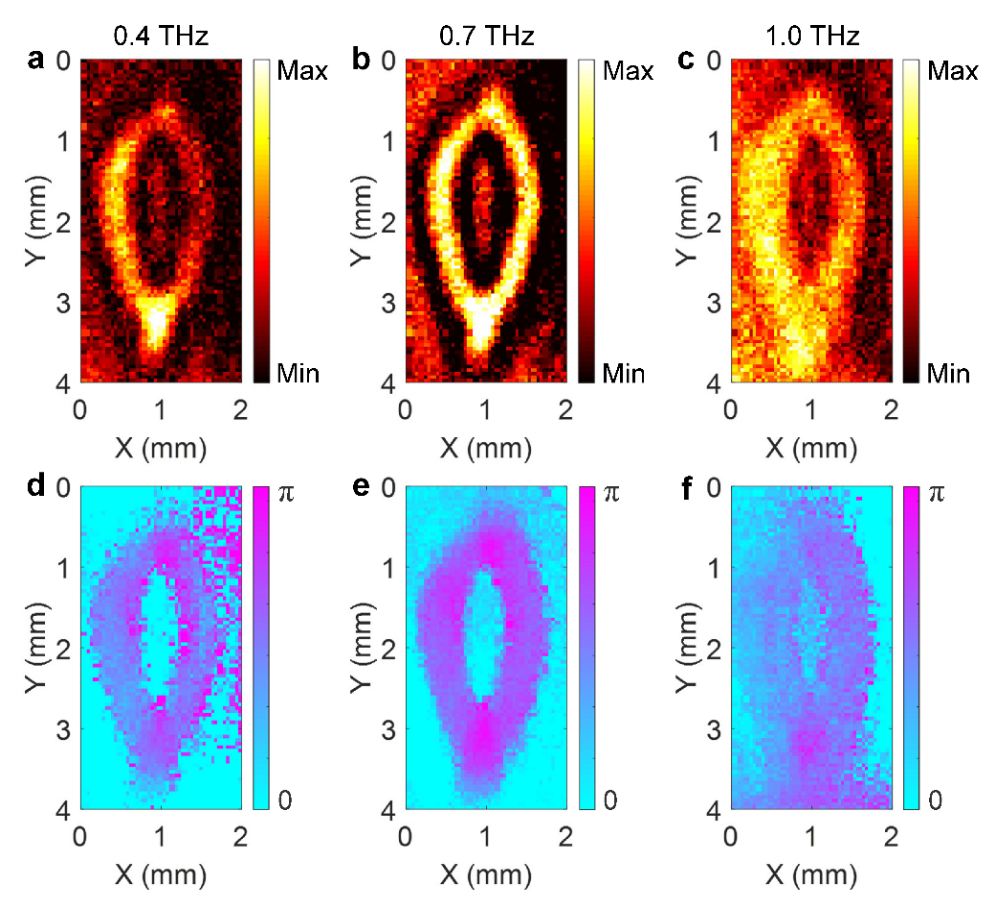
**

**Figure S13| THz intensity and phase images of a plastic pattern. a, b, c,** THz intensity images of the petal pattern acquired at 0.4 THz, 0.7 THz, and 1.0 THz, respectively. **d, e, f,** Corresponding THz phase images.

**IV-b. THz intensity and phase images of a greasy spot**

**
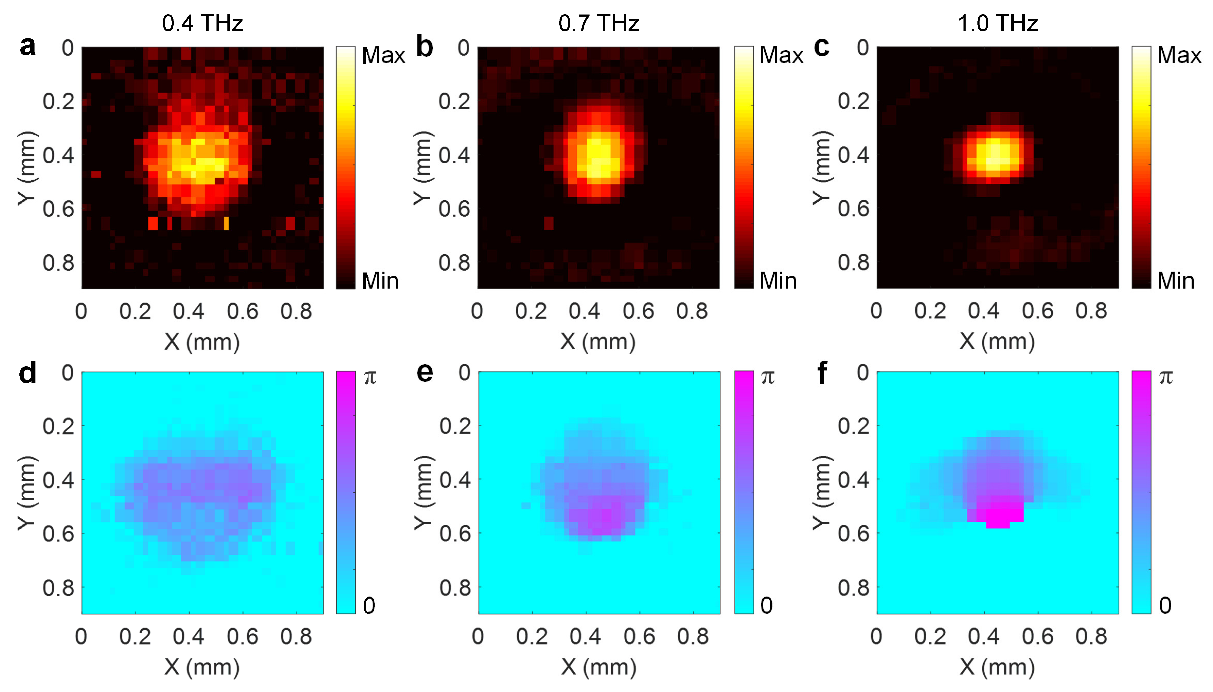
**

**Figure S14| THz intensity and phase images of a greasy spot. a, b, c,** THz intensity images of the greasy spot acquired at 0.4 THz, 0.7 THz, and 1.0 THz, respectively. **d, e, f,** Corresponding THz phase images.

**V Discussion about improving the resolution**

**
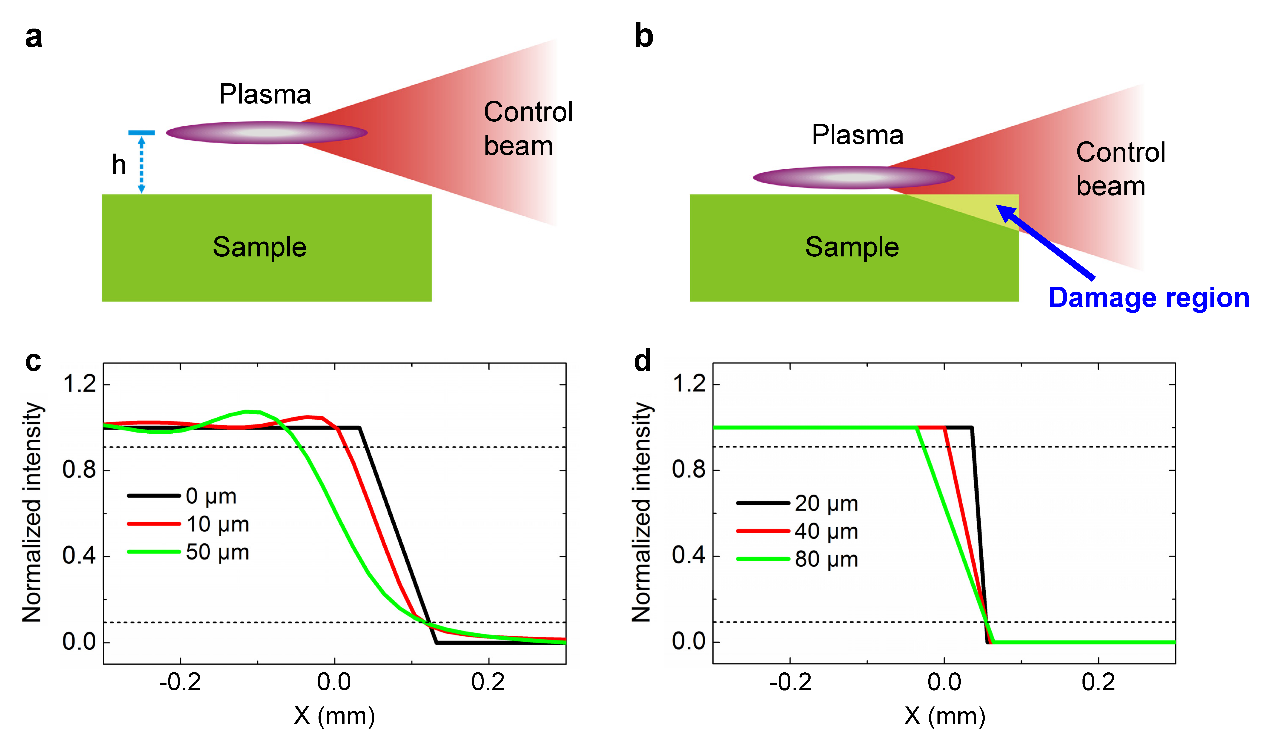
**

**Figure S15| Analysis of improving the resolution. a, b,** Limit of adjusting the height *h* of the cross-filament above the sample surface, including cases without (a) and with (b) sample damage. **c,** Normalized simulated resolution curves with *h*=0 µm, 10 µm, 50 µm for 1.35 THz. The side length of the square detector was fixed at 80 µm. **d,** Normalized simulated resolution curves for 1.35 THz when the side length of the detector was adjusted to 20 µm, 40 µm, 80 µm. The height *h* was fixed at 0 µm.

If we want to obtain a smaller spatial resolution, we have to reduce the height *h* of the cross-filament as far as possible. However, sample damage is caused when *h* is beyond a limit. As shown in Fig. S15a, when *h* is large enough, the sample is not touched by the control beam and is safe. When *h* is excessively reduced and the sample is irradiated by the control beam, sample damage is caused and the filament morphology is also influenced, as shown in Fig. S15b. In our opinion, there is not a specific criterion about the limit of *h*. It is strongly dependent on the NA of a lens and the size of a sample. When a lens with large NA is used to focus the control beam, it is hard to achieve a small *h*. In the same way, when the sample size is too large, the measurement of its central region is hard to be implemented. In this work, the size of the Cr film was only 1 mm×1 mm, so the height *h* could reach 50 μm without damaging the sample. The size of the semiconductor chip was 5 mm×5 mm, so *h* was adjusted to 100 μm. In addition, the imaging regions were near the edges of the chip. About the plastic pattern and the greasy spot, they had larger sizes and uneven surfaces, so *h* was adjusted to 200 μm for avoiding sample damage.

To analyze the limit case, we simulated the normalized resolution curves with *h*=0 µm, 10 µm, 50 µm for 1.35 THz by using the aperture-transmission model, as shown in Fig. S15c. The side length of the square detector was fixed at 80 µm. The resolutions with *h*=0 µm, 10 µm, 50 µm are 80 µm, 106 µm, 152 µm, respectively. It could be seen that the resolution without the diffraction effect matched with the side length of the detector (corresponding to the filament diameter). With increasing *h*, the resolution was gradually deteriorated by the diffraction effect. We also simulated the normalized resolution curves with different side lengths of the square detector. Fig. S15d shows the simulation results for 1.35 THz when the side length of the detector was adjusted to 20 µm, 40 µm, and 80 µm. The height *h* was fixed at 0 µm. The resolutions are 20 µm, 40 µm, and 80 µm, respectively. It could be seen that the resolution can be improved by reducing the size of the detector (corresponding to the filament size). All resolutions without the diffraction effect matched with the corresponding filament sizes. However, the specific method of reducing the filament size is still unclear. Actually, the density inside a filament is non-uniform. The model of the cross-filament is relatively simple in our simulation. As mentioned in the section III-d, the resolutions for 0.1 mJ, 0.2 mJ, and 0.3 mJ pulse energies were almost the same. In addition, the resolutions for 0.4 mJ and 0.5 mJ pulse energies were better. Therefore, the problem needs to be investigated further. We believe that the height *h* and the filament size can be further reduced and the resolution can be further improved. For example, we can used diffraction-free beams (Bessel beam, Airy beam, etc.) as the control beams to generate the cross-filament, which possibly ensure a smaller *h*. In addition, we can used phase-retrieval algorithms (Gerchberg-Saxton, Yang-Gu, etc.) to design special wave-front modulators for shaping the control beam and reducing the filament size. In summary, there is much room left for improving the near-field technique.

**References**

1. Löffler T., Hahn T., Thomson M., Jacob F., & Roskos H. G. Large-area electro-optic ZnTe terahertz emitters. *Opt. Express* **13,** 5353-5362 (2005).
2. Wu Q., Litz M., & Zhang X.-C. Broadband detection capability of ZnTe electro-optic ﬁeld detectors. *Appl. Phys. Lett.* **68,** 2924-2926 (1996).
3. Aschaffenburg D. J. et al. Efficient measurement of broadband terahertz optical activity. *Appl. Phys. Lett.* **100,** 241114 (2012).
4. Yang Y. H., Shutler A., & Grischkowsky D. Measurement of the transmission of the atmosphere from 0.2 to 2 THz. *Opt. Express* **19,** 8830-8838 (2011).
5. Zheng Z. G. et al. Filament characterization via resonance absorption of terahertz wave. *Phys. Plasmas* **24,** 103303 (2017).
6. Goodman J. W. (eds) Introduction to Fourier Optics (New York, USA: McGraw-Hill press, 1996).
7. Gillen G. D. & Guha S. Modeling and propagation of near-ﬁeld diffraction patterns: A more complete approach. *Am. J. Phys.* **72,** 1195-1201 (2004).
8. Deng Y. P. et al. Transverse evolution of a plasma channel in air induced by a femtosecond laser. *Opt. Lett.* **31,** 546-548 (2006).
9. Liu W. & Chin S. L. Direct measurement of the critical power of femtosecond Ti:sapphire laser pulse in air. *Opt. Express* **13,** 5750-5755 (2005).
10. Murzanev A. et al. Superfilamentation in air reconstructed by transversal interferometry. *Phys. Rev. A* **100,** 063824 (2019).
11. Wu T. et al. Excitation-wavelength-dependent terahertz wave modulation via preformed air plasma. *Appl. Phys. Lett.* **112,** 171106 (2018).
12. Bodrov S. et al. Plasma filament investigation by transverse optical interferometry and terahertz scattering. *Opt. Express* **19,** 6829-6835 (2011).
13. Stantchev R. I. et al. Compressed sensing with near-field THz radiation. *Optica* **4,** 989-992 (2017).
14. Stantchev R. I. et al. Noninvasive, near-field terahertz imaging of hidden objects using a single-pixel detector. *Sci. Adv.* **2,** e1600190 (2016).
